# Effect of Sn Content in a CuSnZn Metal Precursor on Formation of MoSe_2_ Film during Selenization in Se+SnSe Vapor

**DOI:** 10.3390/ma9040241

**Published:** 2016-03-29

**Authors:** Liyong Yao, Jianping Ao, Ming-Jer Jeng, Jinlian Bi, Shoushuai Gao, Guozhong Sun, Qing He, Zhiqiang Zhou, Yun Sun, Liann-Be Chang

**Affiliations:** 1 Institute of Photoelectronic Thin Film Devices and Technology and Tianjin Key Laboratory of Thin film Devices and Technology, Nankai University, Tianjin 300071, China; yaoliyong@yeah.net (L.Y.); bijinlian815@126.com (J.B.); gaoshoushuai@yeah.net (S.G.); taigic@nankai.edu.cn (G.S.); Heqing27@nankai.edu.cn (Q.H.); zhqzhou@126.com (Z.Z.); suny@nankai.edu.cn (Y.S.); 2 Department of Electronic Engineering, Chang Gung University, Taoyuan City 33302, Taiwan; liann@mail.cgu.edu.tw

**Keywords:** Cu_2_ZnSnSe_4_ solar cells, selenization, electrodeposited CuSnZn precursor, MoSe_2_, Se+SnSe*_x_* vapor

## Abstract

The preparation of Cu_2_ZnSnSe_4_ (CZTSe) thin films by the selenization of an electrodeposited copper–tin–zinc (CuSnZn) precursor with various Sn contents in low-pressure Se+SnSe*_x_* vapor was studied. Scanning electron microscope (SEM) and energy dispersive spectroscopy (EDS) measurements revealed that the Sn content of the precursor that is used in selenization in a low-pressure Se+SnSe*_x_* vapor atmosphere only slightly affects the elemental composition of the formed CZTSe films. However, the Sn content of the precursor significantly affects the grain size and surface morphology of CZTSe films. A metal precursor with a very Sn-poor composition produces CZTSe films with large grains and a rough surface, while a metal precursor with a very Sn-rich composition procures CZTSe films with small grains and a compact surface. X-ray diffraction (XRD) and SEM revealed that the metal precursor with a Sn-rich composition can grow a thicker MoSe_2_ thin film at CZTSe/Mo interface than one with a Sn-poor composition, possibly because excess Sn in the precursor may catalyze the formation of MoSe_2_ thin film. A CZTSe solar cell with an efficiency of 7.94%was realized by using an electrodeposited metal precursor with a Sn/Cu ratio of 0.5 in selenization in a low-pressure Se+SnSe*_x_* vapor.

## 1. Introduction

Earth-abundant and non-toxic light absorber materials, such as Cu_2_ZnSn(S*_x_*Se_1−*x*_)_4_ (CZTSSe), are attractive for use in the Tera-Watt scale production of thin film solar cells that have adjustable bandgaps of between 1.0 to 1.5 eV and a theoretical efficiency of 30% [1,2,3,4,5,6,7,8,9,10,11,12]. These materials are still in an incipient state of development, and the Cu_2_ZnSnSe_4_ (CZTSe) and CZTSSe solar cells have a maximum device efficiency of 11.6% [4] and 12.6% [5], respectively. The selenization of metal precursors has been demonstrated to be a promising method for large-area and high-efficiency CZTSe solar cells [11,12]. A precursor, wholly [4,5,10] or partially [11,12,13,14] with CZTSe, was first prepared via vacuum or non-vacuum method and then was selenized by thermal treatment to improve its crystallinity. An inert gas and selenium (Se) at high partial pressures are commonly used to ensure the surface stability at the expense of the diffusion of excess Se diffusing through CZTSe thin films, which then reacts with molybdenum (Mo) to form thick molybdenum diselenide (MoSe_2_) films [15].These thick MoSe_2_ films detrimentally affect high-efficiency CZTSe solar cells. Reducing the formation of MoSe_2_ films is a matter of great interest. Severe tin (Sn) loss is commonly observed during high-temperature selenization in the preparation of CZTSe films [16,17,18]. This critical problem arises mainly from the decomposition of CZTSe films. Excess Sn in the precursor is commonly compensates the loss of Sn [19]. However, a thick MoSe_2_ film at CZTSe/Mo interface is observed following the selenization of the metal precursor that contains excess Sn [13]. Shin [15], and Scragg [20] studied the mechanism of formation of the MoSe_2_ film. A high Se concentration in the selenization atmosphere causes Se to diffuse through the CZTSe thin film and react with Mo to form a MoSe_2_ film. However, the mechanism of formation is used only with a near-stoichiometric composition [3,21]. The effect of the Sn content of the precursor on the formation of MoSe_2_ films has not yet been studied. This study investigates CZTSe films that are prepared by the selenization of the metal precursor with various Sn contents in low-pressure Se+SnSe vapor atmospheres, with a focus on the relationship between the Sn content and the formation of MoSe_2_ films.

## 2. Experimental Section

Electrodeposited thin metal stacks were used to fabricate CZTSe thin films by selenization. The cathode in the electrodeposition process was a 4 cm × 4 cm piece of soda-lime glass on which a 1200-nm-thick double molybdenum (Mo) layer was deposited by DC magnetron sputtering. Before the Mo thin films were deposited, the substrates were ultrasonically cleaned in a detergent bath, followed by acetone, isopropanol, and DI water. The DC power was then set at 450 W at room temperature during sputtering. Next, Mo bilayer films were deposited to obtain good adhesion and low resistivity. Mo film was first deposited in a high working pressure of 1.5 Pa for 13 min to obtain good adhesion and then in a low working pressure of 0.1 Pa for 39 min to obtain a low resistivity. The metal stacks of copper (Cu), tin (Sn), and zinc (Zn) were electrodeposited on a Mo layer by the constant current method using a three-electrode system at room temperature. The stacking sequence of the three metals was copper-tin-zinc (CuSnZn) with the Cu layer at the bottom and adjacent to the Mo layer, the Sn layer in the middle, and the zinc layer on the top surface. Table 1 presents the elemental contents of electrodeposited CuSnZn precursors. The ratio of Zn to Cu in electrodeposited CuSnZn precursors was fixed at approximately 0.7. The three ratios of the amount of Sn to the amount of Cu in the electrodeposited CuSnZn precursors were 0.25, 0.5, and 0.75; the corresponding samples were named as A1, A2, and A3, respectively. The proportions of Cu, Sn, and Zn were controlled by adjusting the electroplating times of copper, tin, and zinc. The reference electrode and anode were electrically linked to each other. Copper was electrodeposited from a laboratory-made solution and contained 187.5 g/L of sulfate pentahydrate. Tin was electrodeposited from a commercially available electrodeposition bath, which contained mesylate, tin methanesulfonate, and additives RX-851 from Rongxing Electronics (Zhejiang, China). The solution of zinc was developed in-house using 0.2 M zinc vitriol that was dissolved in 0.5 M methane sulfuric acid. The pH of the solution was adjusted to 2.0 by adding sodium hydroxide. The copper, tin, and zinc layers were electrodeposited using a direct current with current densities of 50, 3, and 20 mA/cm^2^, respectively. The typical deposition time was approximately 100 s.

**Figure 1:**
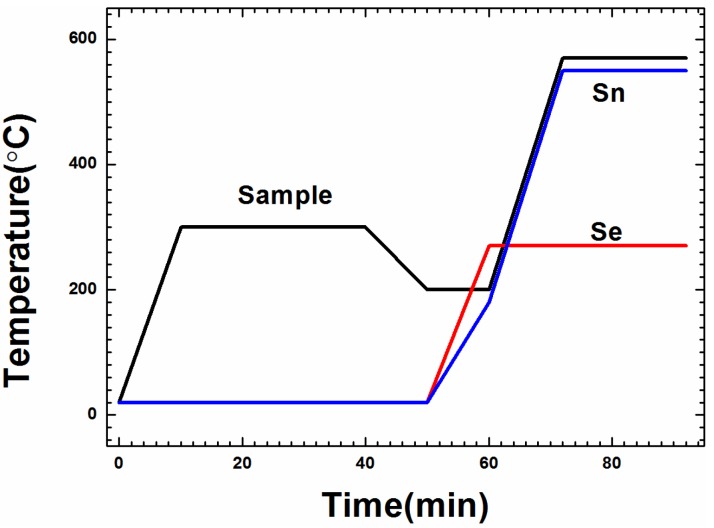
The temperature profiles of the selenization process.

The electrodeposited metal stacks were annealed at 300 °C for 30 min in inert gas at 1000 Pa to ensure the uniform distribution of Cu, Sn, and Zn; they then underwent the selenization processes, as presented in Figure 1. Samples of the metallic precursors were reactively selenized in a laboratory-made furnace (not a tubular furnace) capable of working in vacuum (10^−4^ Pa) or an inert gas (Ar) atmosphere [22]. The sample temperature, Sn temperature and the Se temperature are controlled individually. The temperature of Sn, Se, and sample were at 550, 270, and 570 °C, respectively. The partial pressure of Se and SnSe*_x_* vapors was approximately 16 and 19 Pa, respectively. The elemental Sn and Se are placed in the selenization furnace, under heating, and Se evaporates into the selenization furnace and reacts with Sn to form SnSe*_x_* binary phases, which evaporates easily due to a high saturated vapor pressure. A standard procedure, without any etching process, was applied to fabricate a CZTSe solar cell with a Mo/CZTSe/CdS/i-ZnO/Al-ZnO/Ni-Al structure. First, a 50-nm-thick CdS was deposited by chemical bath deposition. Next, 50 nm i-ZnO and 450 nm Al-ZnO were deposited by RF sputtering. Finally, a 50-nm Ni/0.2-μm Al metal grid was deposited on top of the device via electron-beam evaporation through a metal mask to create a metallic grid pattern. The active area of CZTSe solar cell is 0.34 cm^2^.

The composition of the CZTSe thin film was obtained using a Magix (PW2403 (PANalytical LTD., EA Almelo, The Netherlands)) X-ray fluorescent spectrometer (XRF) with a Rh-anode, which was calibrated by inductively coupled plasma spectroscopy (ICP). The structures of the samples were characterized by using a Philips X-pert Pro diffractometer (PANalytical Ltd.) with Cu Kα radiation and a Renishaw in Via Raman spectroscopy (Renishaw Ltd., Gloucestershire, UK) with an excitation wavelength of 514 nm. The penetration depth of this laser wavelength is around 100 nm into the absorber [23]. Surface and cross-sectional observations were made using a scanning electron microscope with energy dispersive spectroscopy (EDS) analysis. (SEM, JEOL JSM-6700 (JEOL Ltd., Akishima-shi, Japan). The depth profiles of the elements were obtained by line scanning EDS and secondary ion mass spectroscopy (SIMS) measurements (IMS-4F, CAMAECA, Nancy, France). The incident electron probe size used in EDS measurement was 10 nm (full-width-at-half-maximum). The surface roughness was determined by using an Ambios Technology XP-2 Surface Profiler. Current-voltage (J-V) measurements of CZTSe solar cells were made under illumination by a standard AM1.5 spectrum of 1000W/m^2^ at room temperature with a constant-light solar simulator, which was calibrated using a standard mono-crystalline Si solar cell.

## 3. Results and Discussion

**Figure 2:**
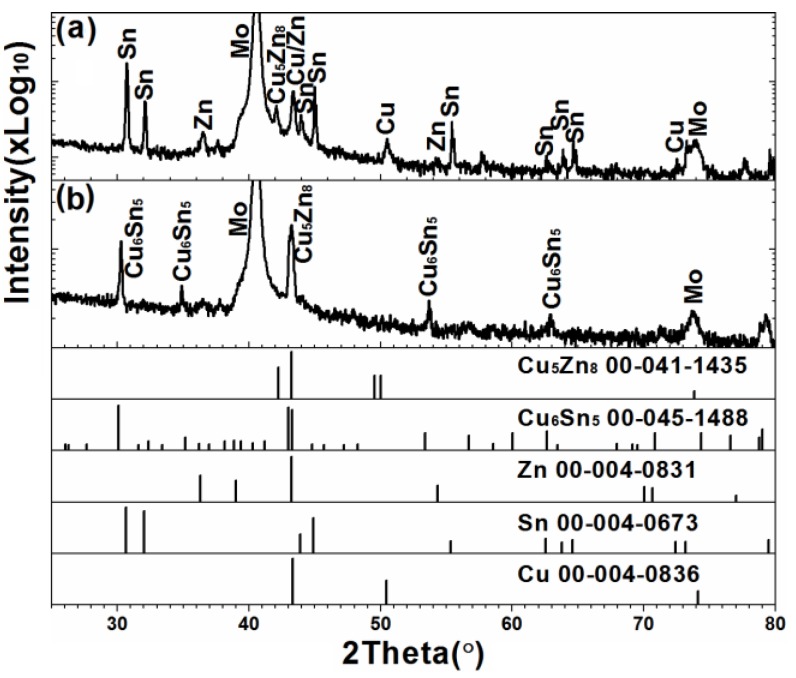
The X-ray diffraction (XRD) patterns of the electrodeposited CuSnZn precursor (**a**) before; and (**b**) after annealing at 300 °C for 30 min.

Figure 2 present the XRD patterns of the electrodeposited CuSnZn precursorbefore and after annealingat 300 °C for 30 min, respectively.The XRD spectra in Figure 2a included strong Cu, Sn, and Zn elemental peaks and a weak Cu_5_Zn_8_ alloy peak. Annealing eliminated the Cu, Sn, and Zn elemental peaks and caused the appearance of strong peaks that were associated with Cu_5_Zn_8_ and Cu_6_Sn_5_ alloys in Figure 2b. High-temperature annealing promoted the interdiffusion of elements to form a binary alloy in the metallic stack precursor. The adhesion between CZTSe and Mo was a critical problem in the selenization of the metal precursor in the preparation of CZTSe thin films. Stacked metal layer, especially with the Cu at the bottom, cause bad adhesion between CZTSe films and Mo substrates. The volume of the copper clearly varies during the selenization process, owing to the large disparities between the volume of Cu_2_Se and CuSe_2_ per one copper atom [24]. The bottom Cu may generate holes at the back contact interface and thereby form many point defects during selenization, detrimentally affecting device performance [14]. After annealing, the formation of the binary alloy in the metallic stack precursor may reduce the number of pin holes and defects formed. Formation of alloy in the precursor favors follow-up selenization for the preparation of high-quality CZTSe films.

Table 2 presents the elemental contents of the CZTSe films that were prepared by the selenization of the precursor with samples A1, A2, and A3 in a SnSe*_x_*+Se vapor atmosphere. The metal ratio of Zn to Cu in all three samples after selenization is 0.69. As is well known, the Zn has a high saturated vapor pressure at temperatures of over 400 °C [25]. However, during selenization, Zn easily reacts with Se to form a ZnSe compound, which has a low saturated vapor pressure. Therefore, the Zn does not easily evaporate during selenization. Interestingly, the ratio of Sn to Cu varies only slightly between 0.62 and 0.65, even as the Sn/Cu ratio in the precursor is greatly increased from 0.25 to 0.75. A large change in the Sn content of the metal precursor after selenization results in a small change in the Sn content of the formed CZTSe films, mainly owing to the following chemical reaction [16,17,18].

Cu_2_ZnSnSe_4_ ↔ Cu_2_Se + ZnSe + SnSe_(g)_ + 1/2S(e)_2(g)_ (1)

The reaction will proceed toward CZTSe (turn to the left reaction) when the Se partial pressure is very high, especially when accompanied by a high inert gas pressure. However, the decomposition reaction of CZTSe thin films (turn to the right reaction) will occur when the Se partial pressure or inert gas pressure is not high enough. In addition, The CZTSe thin film is stable when SnSe and Se coexist in the selenization atmosphere, which has been confirmed by experiments [17,18]. In Table 2, a large change in the Sn content of the metal precursor after selenization results in a small change in the Sn content of the formed CZTSe films, implying that the reaction is a self-regulating process during the selenization in SnSe and Se atmosphere. When the metal precursor is Sn-rich, the right-hand side of the above chemical reaction (turn to the right reaction) is favored and the excess Sn evaporates off as SnSe_(g)_. In contrast, when the metal precursor is Sn-poor, the left-hand side of the chemical reaction (turn to the left reaction) is favored and the SnSe*_x_*_(g)_ in the atmosphere reacts with the Se-related containing phases Cu_2_Se, ZnSe, and Se to form CZTSe film. Therefore, selenization only slightly changes the Sn content, as presented in Table 2. Similar results have been obtained elsewhere [18].

**Figure 3:**
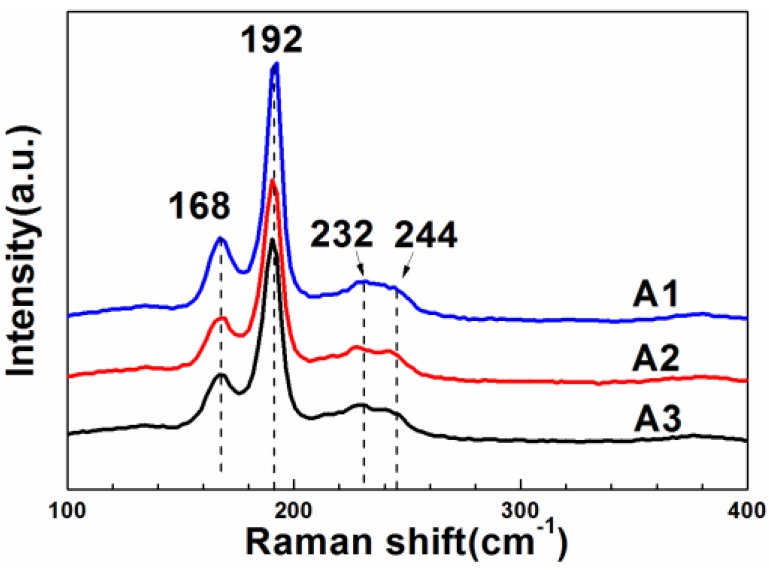
The Raman spectra of the CZTSe films that were prepared by the selenization of the precursor with samples A1, A2, and A3.

Figure 3 presents the Raman spectra of the CZTSe films that were prepared by the selenization of the precursor with samples A1, A2, and A3. Peaks at 168, 192, 232, and 244 cm^−1^, associated with the CZTSe phase, were obtained from all three samples. The Raman patterns of the three samples are very similar, indicating that the surface phases of these CZTSe films were similar because the penetration depth of Raman measurement is around 100 nm into the absorber [23]. The similarity of the phase structures of the three samples arises mainly from the use of a single selenization atmosphere of Se+SnSe*_x_* vapor. According to the chemical reaction (1), the same reactive selenization atmosphere will result in the same CZTSe phases. The lack of any MoSe_2_-related peaks from any of the three samples does not reveal the absence of MoSe_2_ because the Raman measurements could only be made to a depth of approximately 100 nm with an exciting laser having a wavelength of 514 nm, whereas the thickness of CZTSe films was approximately 2 μm. Therefore, Raman measurement could not detect MoSe_2_ films at CZTSe/Mo interface. Accordingly, other measurements, such as XRD or SIMS, had to be made to identify the formation of MoSe_2_.

**Figure 4:**
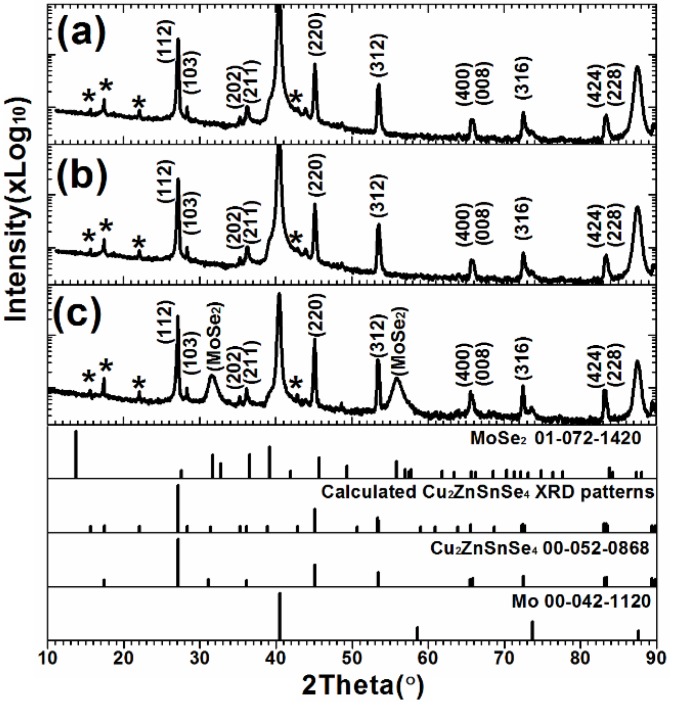
The XRD spectra of the CZTSe thin films that were prepared by the selenization of the precursor with samples (**a**) A1; (**b**) A2; and (**c**) A3.

Figure 4 presents the XRD spectra of the CZTSe thin films that were prepared by the selenization of the precursor with samples A1, A2, and A3. The CZTSe phases are identified by standard XRD pattern (JCPDS 00-052-0868), based on experimental data. Additional CZTSe diffraction peaks, labeled as * (calculated to correspond to a body-centered tetragonal structure of lattice constants a = 5.693 Å and c = 11.333 Å) are also identified. The XRD patterns of the three samples are similar, revealing that they have similar structures. However, sample 3 yielded two strong peaks at 31.6° and 55.9°, which correspond to MoSe_2_ structures, indicating that the selenization of metal precursor with excess Sn content may promote the formation of a MoSe_2_ layer at the CZTSe/Mo interface. The CZTSe (112) peak position of sample A1, A2, and A3 was 27.14°, 27.17°, and 27.27°, respectively. However, the FWHM of the CZTSe (112) peak was the same value of 0.1299 for the three samples. It cannot observe any difference of grain size from the XRD results of the three samples. According to the standard XRD peaks of CZTSe (00-052-0868) and ZnSe (00-037-1436), the ZnSe (111) peak position has a larger angle than the CZTSe (112) peak position, implying that the ZnSe secondary phases in the three samples may be different.

**Figure 5:**
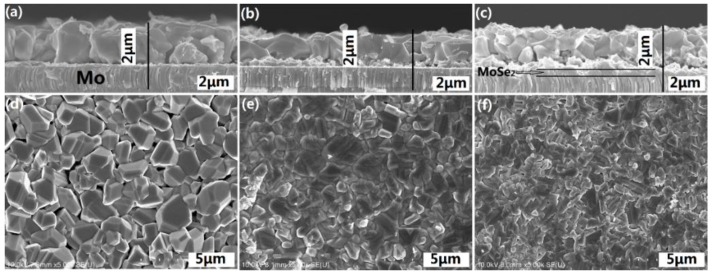
The surface morphology and cross-sectional SEM images of the CZTSe films that wereprepared bythe selenization of the precursor with samples (**a**,**d**) A1;(**b**,**e**) A2; and (**c**,**f**) A3.

**Figure 6:**
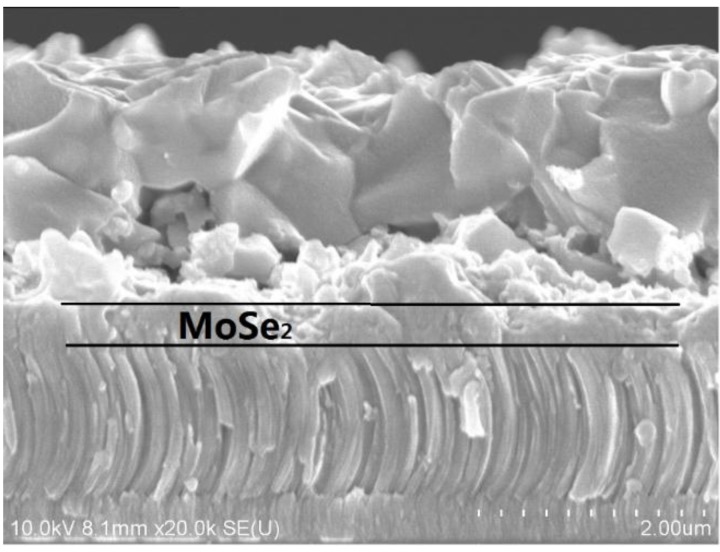
The magnification SEM image of Figure 5c.

To confirm the formation of a MoSe_2_ thin film at CZTSe/Mo interface, Figure 5 presents the surface morphology and cross-sectional SEM images of the CZTSe films that were prepared by the selenization of the precursor with samples A1, A2, and A3. No MoSe_2_ layers are observed in Figure 5a,b. However, the MoSe_2_ film is hardly recognizable in Figure 5c. The magnification SEM image of Figure 5c is shown in Figure 6. A clear MoSe_2_layer was observed, consistent with the XRD observation in Figure 4c. The MoSe_2_ thin film formed at the CZTSe/Mo interface when the metal precursor contained excess Sn. The SnSe-containing phase (SnSe_2_) may have catalyzed the formation of MoSe_2_ thin film [26]. The Gibbs free energy changes for the chemical reactions of 2Se + Mo = MoSe_2_ and 2SnSe_2_ + Mo = 2SnSe + MoSe_2_ are calculated by the chemical software of enthalpy, entropy and heat capacity (HSC) 5.0 (Outouec Ltd., Pori, Finland). The Gibbs free energy changes of the two reactions are negative, implying that both of the chemical reactions may occur spontaneously. However, in our previous study [22], the formation of MoSe_2_ layer is very slow when the selenization of metal precursor is at a low Se partial pressure atmosphere. Therefore, we can conclude that the formation of the MoSe_2_ layer in sample A3 is dominated by the reaction of 2SnSe_2_ + Mo = 2SnSe + MoSe_2_, owing to the excess of SnSe*_x_* from the reactions of Cu*_x_*Sn*_y_* + 2Se*_x_*_(g)_ → Cu_2−*x*_Se + SnSe*_x_* during selenization. The three CZTSe films had very different surface morphologies and cross-sectional images.The CZTSe films that were prepared by sample A1 (very Sn-poor CuSnZn precursor) contained large grains, as presented in Figure 5d with many large gaps or holes at the grain boundaries. From the equilibrium phase diagram, there exists a very samll region for the formation of a single phase CZTSe thin film [27]. No Cu-Zn-Se and Sn-Zn-Se ternary phases can form in CZTSe films. In sample A1, the CuSnZn precursor was very Cu-rich and Sn-poor. The ZnSe, Cu_2−*x*_Se and CZTSe phases co-existed in the film during the intial annealing process. Cu_2−*x*_Se is liquid phase at a temperature of 570 °C, effectively promoting the peritectic reaction and grain growth [28]. However, in sample A3 (very Sn-rich CuSnZn precursor) in Figure 5c,f, the grains are small, and the surface morphology is compact. Many secondary phases are present at the CZTSe/Mo interface. The Cu_2−*x*_Se is quickly consumed by the reaction (2) with the SnSe*_x_* phase to form Cu_2_SnSe_3_ phase in the intial selenization. The CZTSe thin film is formed by the solid reaction (3), and small grains are therefore obtained.

Cu_2−*x*_Se + SnSe_2_ → Cu_2_SnSe_3_ (2)

Cu_2_SnSe_3_ + ZnSe = Cu_2_ZnSnSe_4_ (3)

**Figure 7:**
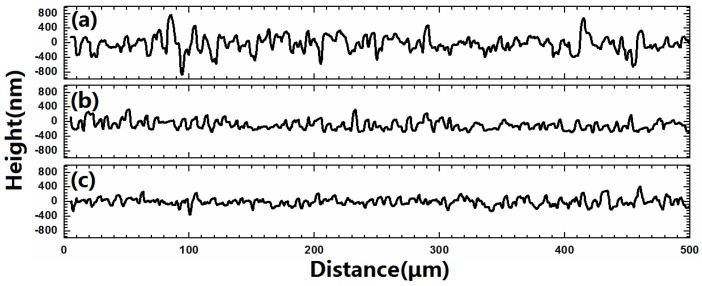
(**a**–**c**) present the two dimensional surface profiles of the CZTSe films that correspond to Figure 5d–f, respectively.

**Figure 8:**
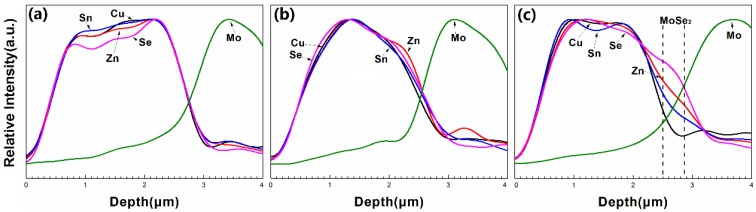
The line scanning energy dispersive spectroscopy (EDS) depth profiles of the CZTSe films that were prepared bythe selenization of the precursor with samples (**a**) A1; (**b**) A2; and (**c**) A3.

**Figure 9:**
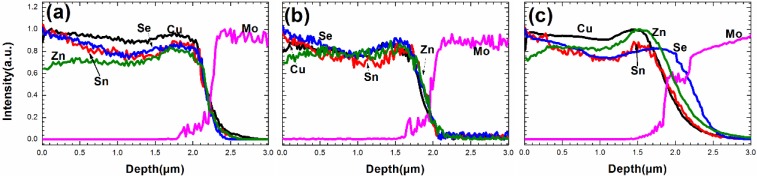
The secondary ion mass spectroscopy (SIMS) depth profiles of the CZTSe films that were prepared by the selenization of the precursor with samples (**a**) A1; (**b**) A2; and (**c**) A3.

Figure 7 presents the two dimensional surface profiles of the CZTSe films that correspond to Figure 5d–f, respectively. The root-mean-square (RMS) roughness for samples A1, A2, and A3 are 245.8, 122.3, and 105.8 nm, respectively. A high rough surface was obtained when the metal precursor was Sn-poor, consistent with the SEM observations in Figure 5. The liquid Cu_2−*x*_Se phase promotes grain growth and forms a rough surface during selenization. Figure 8a–c present the line scanning EDS depth profiles of the CZTSe films that correspond to Figure 5a–c, respectively. Figure 8a presents a uniform element distribution of Cu, Sn, Zn, and Se element from surface to the CZTSe/Mo interface. Figure 8b presents weak Sn, Zn, and Se element signals from the CZTSe/Mo interface, corresponding to a thin interface layer of secondary phases. However, in Figure 8c, clear Sn, Zn, and Se element signal are obtained from the CZTSe/Mo interface, corresponding to a thick interface layer that comprises secondary phases. Figure 9 present the SIMS depth profiles of the CZTSe films that were prepared by the selenization of the precursor with samples A1, A2, and A3, consistent with the line scanning EDS observation in Figure 8. The excess signals of Se and Zn elements from the observation of line scanning EDS or SIMS measurement indicate that ZnSe secondary phases possibly exist at the back contact. The secondary phases of ZnSe at the back contact may introduce a high series resistance and degrade the performance of solar cells [29,30]. The Se signal diffuses deeply into the Mo substrate, indicating that a thick MoSe_2_ layer formed at the CZTSe/Mo interface, consistent with the XRD observation in Figure 4b.

**Figure 10:**
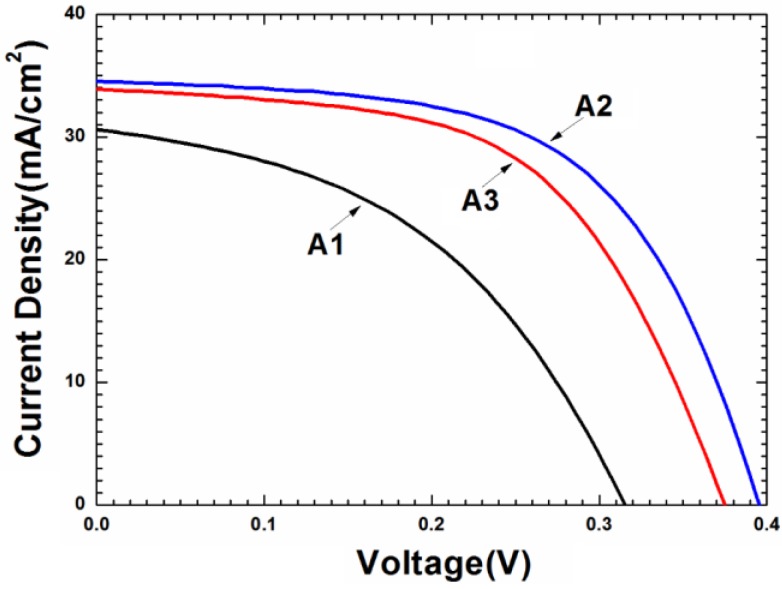
Current-voltage (I-V) curves of three CZTSe solar cells by using films that were formed by the selenization of the precursor with samples A1, A2, and A3.

Figure 10 plots current-voltage (I-V) curves of three CZTSe solar cells by using films that were formed by the selenization of the precursor with samples A1, A2, and A3 (active area: 0.34 cm^2^). Table 3 presents the performance parameters of these three solar cells. The CZTSe solar cell that was prepared by using sample A2 exhibited the best device performance with performance parameters of η = 7.94%, Voc = 0.394 V, Jsc = 34.51 mA·cm^−2^, and FF = 0.584. However, the CZTSe solar cell prepared by sample A1 exhibits the worst performance with performance parameters of η = 4.30%, Voc = 0.316 V, Jsc = 30.59 mA·cm^−2^, and FF = 0.445. Large grain boundaries provide a leakage current path, and the rough surface may form many recombination centers at CZTSe/CdS interface [31], seriously degrading the performance of device that was fabricated by using sample A1. However, small grains and thick MoSe_2_ layer at CZTSe/Mo interface may be responsible for the poor performance of sample A3.

## 4. Conclusions

The effect of Sn content of the CuSnZn precursor on the formation of CZTSe films was studied. Experiments revealed that the Sn content of the precursor in the selenization in a low-pressure vapor atmosphere only slightly influences the elemental composition of CZTSe films. However, the Sn content of the precursor significantly affects the grain size and surface morphology of formed CZTSe films. A Sn-poor metal precursor tends to lead to the growth of a CZTSe film with large grains and a rough surface, whereas a Sn-rich precursor tends to lead to the growth of a CZTSe film with small grains and a compact surface. Additionally, the Sn content of the precursor greatly affects the formation of MoSe_2_thin film: A Sn-rich metal precursor leads to the growth of a thicker MoSe_2_ thin film than does a Sn-poor precursor, because excess Sn is catalyzing. A CZTSe solar cell with an efficienct of 7.94% was realized by using the metal precursor with a Sn/Cu ratio of 0.5 in the selenization process in a low-pressure Se+SnSe*_x_* vapor atmosphere.

## Figures and Tables

**Table 1:** The elemental contents of electrodeposited CuSnZn precursors.

Sample	Cu (%)	Zn (%)	Sn (%)	Zn/Cu	Sn/Cu	Cu/(Zn+Sn)	Zn/Sn
A1	51.28	35.9	12.82	0.70	0.25	1.05	2.80
A2	45.45	31.81	22.74	0.70	0.50	0.83	1.40
A3	40.82	28.57	30.61	0.70	0.75	0.69	0.93

**Table 2:** The elemental contents of the CZTSe films that were prepared by the selenization of the precursor with samples A1, A2, and A3 in a SnSe*_x_*+Se vapor atmosphere.

Sample	Cu (%)	Zn (%)	Sn (%)	Se(%)	Zn/Cu	Sn/Cu	Cu/(Zn+Sn)	Zn/Sn
A1	21.38	14.75	13.24	50.63	0.69	0.62	0.76	1.11
A2	21.02	14.51	13.19	51.28	0.69	0.63	0.76	1.10
A3	19.86	13.74	12.98	53.42	0.69	0.65	0.74	1.06

**Table 3:** Performance parameters of CZTSe solar cells that were prepared by the precursor samples of A1, A2, and A3.

Sample	Eff (%)	Voc (mV)	Jsc (mA·cm^−2^)	FF	Rs (Ω)	Rsh (Ω·cm^2^)
A1	4.30	316	30.59	0.445	3.1	49
A2	7.94	394	34.51	0.584	2.6	265
A3	7.10	376	33.92	0.557	2.72	112
